# High-Resolution Full-Field Structural Microscopy of the Voltage-Induced Filament Formation in VO_2_-Based Neuromorphic Devices

**DOI:** 10.1021/acsnano.4c14696

**Published:** 2025-04-14

**Authors:** Elliot Kisiel, Pavel Salev, Ishwor Poudyal, David J. Alspaugh, Fellipe Carneiro, Erbin Qiu, Fanny Rodolakis, Zhan Zhang, Oleg G. Shpyrko, Marcelo Rozenberg, Ivan K. Schuller, Zahir Islam, Alex Frano

**Affiliations:** † Physics Department, University of California San Diego, La Jolla, California 92093, United States; ‡ X-ray Science Division, Argonne National Laboratory, Lemont, Illinois 60439, United States; § Department Physics and Astronomy, University of Denver, Denver, Colorado 80210, United States; ∥ Materials Science Division, Argonne National Laboratory, Lemont, Illinois 60439, United States; ⊥ Laboratoire de Physique des Solides, CNRS-UMR 8502, Université Paris-Sud, Orsay 91405, France; # Materials Physics and Applications, Los Alamos National Laboratory, Los Alamos, New Mexico 87544, United States; ∇ Centro Brasileiro de Pesquisas Físicas, Rio de Janeiro, RJ 22290-180, Brazil; ○ Program in Materials Science and Engineering, University of California San Diego, La Jolla, California 92093-0418, United States

**Keywords:** full-field diffraction microscopy, dark-field X-ray microscopy, vanadium dioxide, metal−insulator transition, neuromorphic systems, device physics

## Abstract

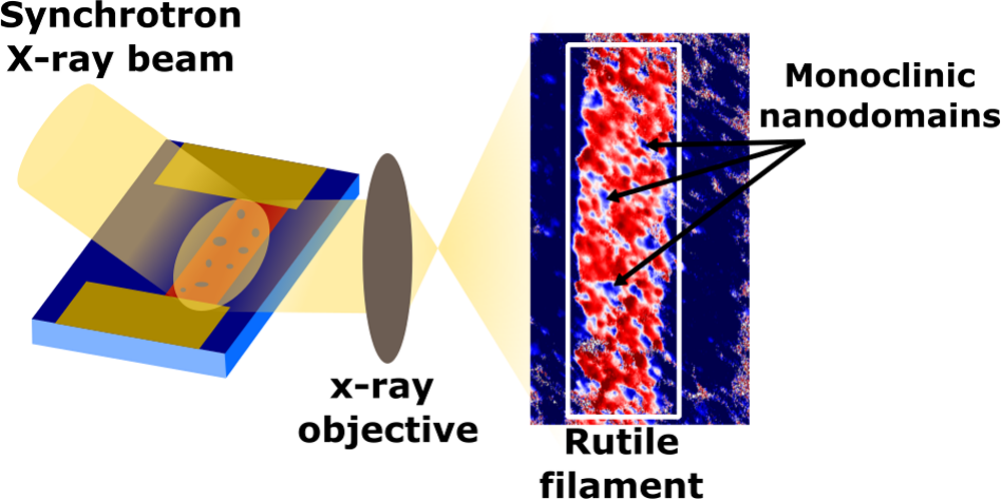

In order to make neuromorphic functions in memristive devices more efficient, information about the structural properties of filament formation at the micro- and mesoscopic scales is necessary. Despite extensive research on VO_2_, a key material due to its filament formation, local operando structural measurements remain challenging and often involve destructive specimen preparation and long rastering times, greatly limiting the scope of experimental studies. Utilizing dark-field X-ray microscopy (DFXM), a full-field imaging modality, structural signatures of the filament formation process operando are revealed in VO_2_ devices. DFXM experiments illustrate that rutile filaments contain isolated monoclinic clusters, indicating structural nonuniformity interior to the filament. The formation of the rutile phase beneath device electrodes was shown to precede filament development, followed by the formation of filament paths guided by nucleation sites within the device. Finally, a medium-term (<30 min) memory mechanism is observed in VO_2_, mediated by sites within the device gap that tend to switch at significantly lower voltages after electrical cycling, a tendency that persists through a brief thermal reset. High spatial resolution, large field-of-view, structure selectivity, and fast signal acquisition of DFXM provided insight into structural features of the filamentary channel and surrounding regions during voltage cycling.

In recent years, significant attention has been paid to the development of neuromorphic hardware for the practical realization of advanced, energy-efficient computational architectures that mimic the complex functionalities of the human brain.^1−5^ The requirements of high scalability and low-power operation of neuromorphic components motivate the search for novel materials beyond semiconductors.^3^ Vanadium dioxide (VO_2_) is a promising material platform for implementing neuromorphic functionalities derived from its electronic and structural properties. VO_2_ undergoes a first-order metal–insulator transition (MIT) coinciding with a monoclinic (M1) to rutile (R) structural phase transition at *T*_C_ ∼ 340 K.^6−9^ Optical, electrical, and mechanical external stimuli can also induce this transition.^10−18^ This tunability allows one to envision practical applications such as building VO_2_-based memristive and signal-processing devices into neuromorphic systems.^2,19−22^

Fundamental to applications is the activation of an MIT using an electrical stimulus that causes VO_2_ to transition to a low-resistance state. The high-resistance state is recovered when the stimulus is turned off.^23^ This resistive switching is due to the electrically induced metallic phase filament formation inside the insulating phase.^10,13,24,25^ The voltage-induced MIT in VO_2_ is most commonly attributed to Joule heating that raises the local temperature above *T*_C_,^26^ although other studies have provided evidence of the importance of nonthermal effects mediated by the electric field.^27^ Ramp-reversal memory in VO_2_, achieved through thermal cycling the VO_2_, have been observed to locally modify transition temperatures of domains presenting possible memory mechanisms in VO_2_ devices.^28,29^ Investigations of filament formation usually probe only the electronic transition, which has left many key questions unanswered.^10,13,25,30^ For example, why do filaments form preferentially in specific locations, or how do the memory/training effects emerge during repeated electrical cycling? The coupling between structural and electronic degrees of freedom in VO_2_ can give rise to exotic phases, such as a metallic monoclinic phase,^31−35^ and thus may play a critical role in electrical switching and relaxation processes. A deeper understanding of the memristive properties, particularly from a structural perspective, is necessary to engineer devices to improve the energy efficiency of VO_2_-based systems.^36^

Structural studies of resistive switching in VO_2_ are often limited in scope. Techniques such as transmission electron microscopy require destructive sample preparation, which imposes limits on the device geometry and size, and may alter local material properties.^23^ Nondestructive techniques such as X-ray micro- and nanodiffraction provide opportunities to explore the structural transition that accompanies the electrical MIT. A previous X-ray nanodiffraction study used coupled MIT and structural transitions to probe electrical switching in VO_2_ devices at a local level.^24^ However, this study was limited to steady-state switched devices (i.e., no voltage cycling) due to the long times needed to raster the full device. This rastering time makes it ineffective to study voltage cycling, limits the field of view (FOV), and prevents investigations of the relaxation processes. To address these challenges and investigate the full device including the electrodes, two key experimental improvements are necessary: (i) an X-ray imaging technique with a large enough FOV to acquire the entire device image in a single exposure, and (ii) a fast image acquisition to improve temporal resolution.

An X-ray imaging technique that meets these requirements is dark-field X-ray microscopy (DFXM). DFXM is a full-field imaging technique using diffraction-intensity contrasts encoded on a Bragg peak to image local mesoscale structural inhomogeneities in materials. Early DFXM studies have been applied to bulk crystals and relatively thick films containing high-Z elements, which enable strong X-ray diffraction signals.^37−39^ VO_2_, however, being composed of low-Z elements, vanadium and oxygen, presents a challenge to DFXM due to its low scattering strength. The scattering signal is further reduced from VO_2_ crystalline thin films due to the reduced material volume. In this work, a practical route is presented that can extend DFXM to systems where the X-ray scattering is weak, for example, scattering from magnetic features or charge density waves.

Here, the structural properties of VO_2_ two-terminal devices are examined during the voltage-induced MIT switching behavior. These experiments revealed three new mesoscale structural phenomena that accompany the MIT switching. First, it is observed that filaments in VO_2_ devices are not structurally uniform and have isolated M1-phase clusters within the R-phase filamentary channel. Second, regions of VO_2_ switch into the R phase underneath the electrodes before the filament formation providing an explanation for filament appearance in specific locations. Finally, the emergence of local clusters with drastically reduced switching voltages was observed following the electrical cycling of the device. These local clusters maintain the memory of the prior MIT triggering events for ∼30 min, even when VO_2_ is cooled to room temperature, effectively resetting the phase state. Upon repeated filament formation, these sites are shown to have a reduced *T*_C_, possibly due to ramp-reversal memory effects. Electrical switching simulations indicated the important role of small local defects with a reduced transition temperature that may be responsible for the switching cycling memory effects, further reinforcing experimental results.

## Results/Discussion

### Dark-Field X-ray Microscopy Application to Thin Films

**Figure 1:**
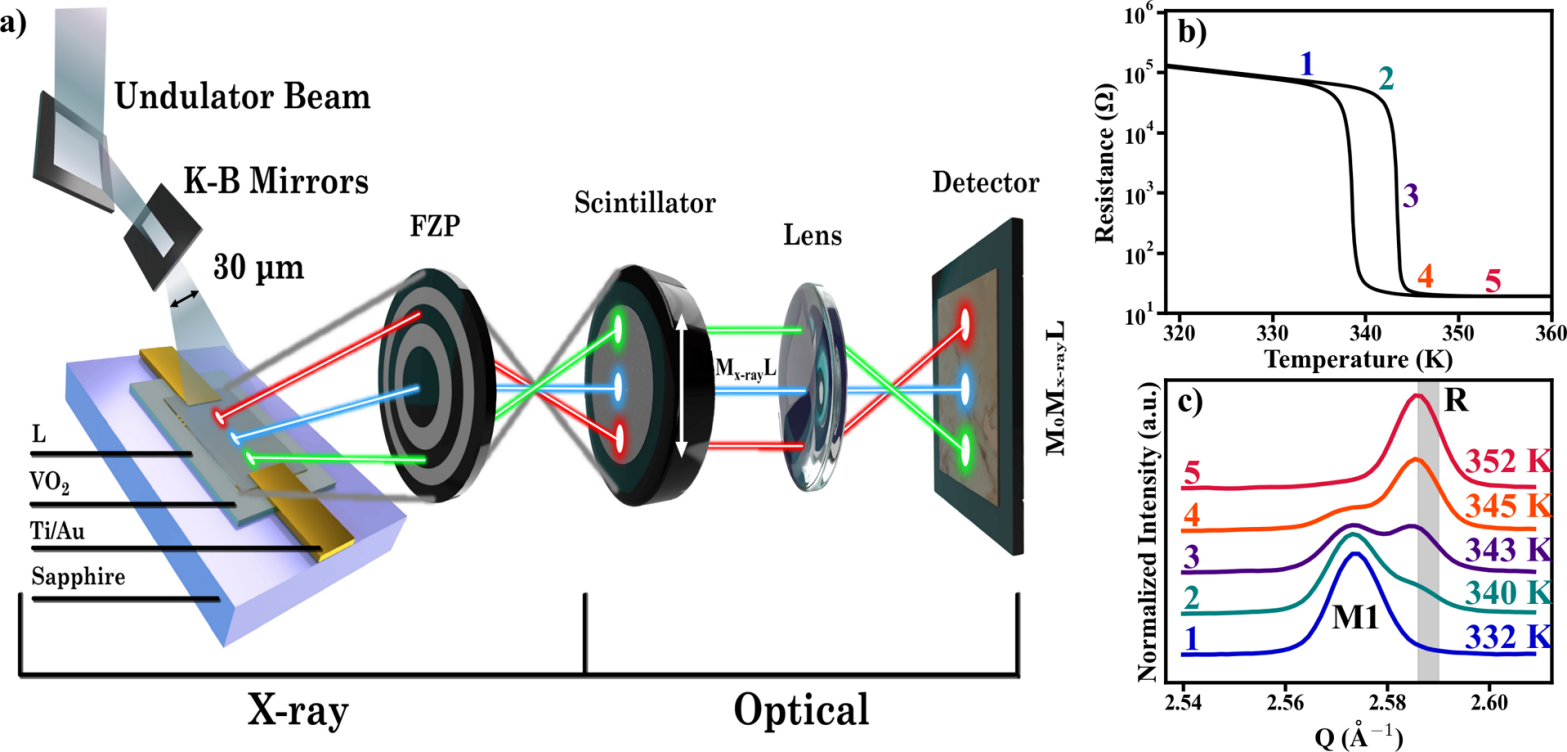
Operation of DFXM and VO_2_ properties. (a) A DFXM setup. The focused beam from the Kirkpatrick–Baez (K–B) mirrors increases the flux density that illuminates the device area. Spatially separated regions of the device (red, blue, and green ovals), when passed through the Fresnel zone plate (FZP), are spatially preserved on the scintillator, giving the magnification of *M*_X-ray_ ∼ 24. An optical lens between the scintillator and detector further magnifies the image by *M*_O_ = 20. The resulting image on the detector for a given sample region *L* is then *M*_X-ray_M_O_L. (b) Resistance–temperature dependence of VO_2_ device. Metal–insulator transition occurs at *T*_C_ ∼ 340 K where the resistance abruptly changes by ∼4 orders of magnitude. (c) Temperature evolution of the structural phase transition from the monoclinic (M1) phase (blue) to rutile (R) phase (red). The shaded gray region marks the scattering condition where the images in Figure 3 were recorded. Numbers 1–5 and associated colors in panels (b) and (c) allow comparisons between electrical and structural data.

Dark-field X-ray microscopy (DFXM) employs the spatially resolved detection of scattered X-rays from a sample, providing enhanced contrast and sensitivity to subtle mesoscopic structural changes.^39−42^ The imaging in DFXM is achieved by placing an X-ray objective lens in the path of the diffracted beam (Figure 1a). Similar to optical microscopy, the objective lens refracts the diffracted X-ray beam producing a magnified device image on the scintillating detector. Unlike optical microscopy which relies on acquiring the reflected light, the contrast in DFXM originates from diffraction; i.e., the images acquired by the detector correspond to spatial variations in the diffraction conditions. The primary advantage of DFXM, as compared to micro- and nanodiffraction techniques, is the ability to acquire large FOV structural images in a single exposure without the need to raster. The full-field imaging of DFXM allows simultaneous acquisition of a large area (30 × 120 μm^2^) with high resolution (<100 nm) of structural signals during filament formation and relaxation in VO_2_ switching devices. Currently, the resolution of the setup is limited by the small numerical aperture of the X-ray objective lens, however, improvements to these lenses may lead to higher resolutions on the order of 10 nm.^43,44^ Moreover, 3D mapping techniques are being investigated to map vertical device geometries allowing access to the investigation of devices typically seen in neuromorphic settings.^45,46^ The fast signal acquisition times afforded by DFXM enabled structural imaging of repeated voltage cycling, which is key to understanding the MIT switching repeatability and emerging memory/training phenomena.

One of the challenges of performing DFXM on VO_2_ is the low intensity of the diffracted beam because of the low-Z element composition. A pair of Kirkpatrick-Baez (K–B) mirrors were used to focus the incident beam to a 30 × 30 μm^2^ spot size to increase the flux density (∼10^13^ photons/s). Stronger incident flux resulted in a larger diffraction intensity, greatly improving the contrast in DFXM images. With the improved incident flux on the sample, images were able to be captured with a field of view of 30 × 120 μm^2^ as fast as 1–10 s. This is in contrast to nanodiffraction imaging, which typically takes multiple hours to collect a single image of a few μm size.^24^ Even with this reduced FOV, the gain in signal strength allowed for observation of the VO_2_ film which was not possible without the increase in flux density.

**Figure 2:**
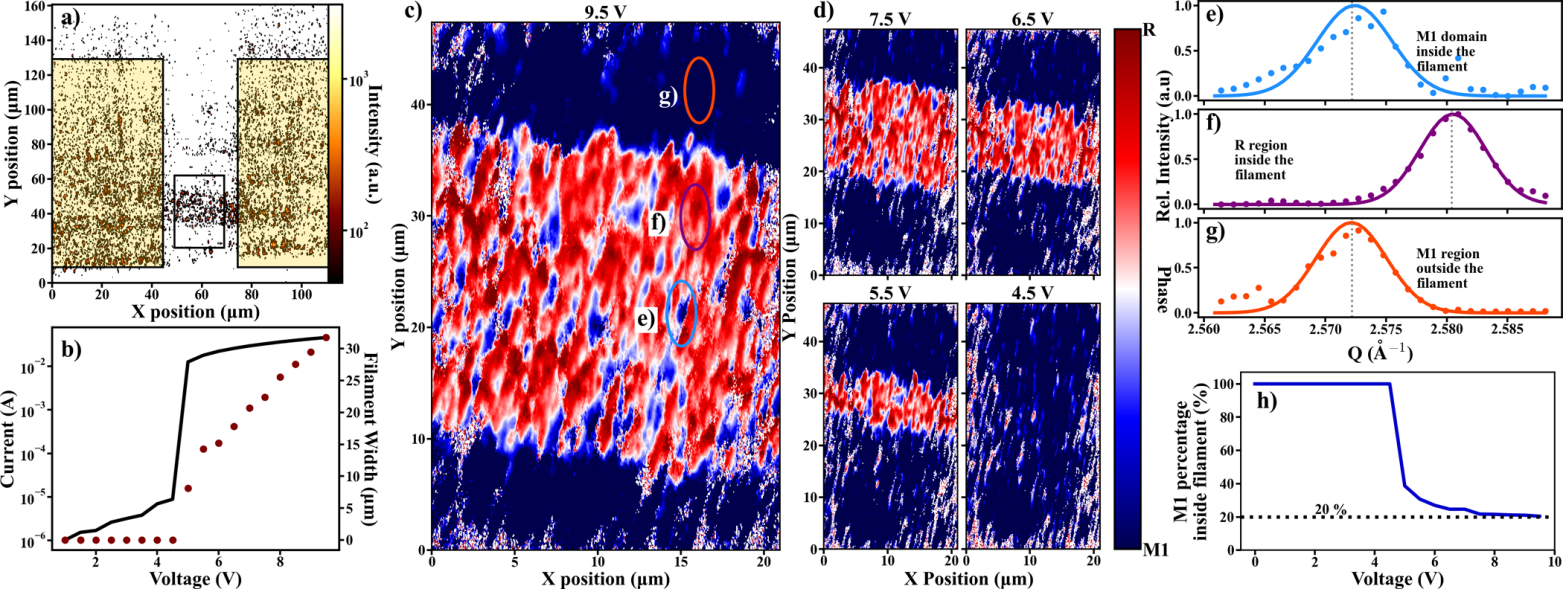
Correlated DFXM imaging and electrical measurements on a VO_2_ device. (a) Rastered DFXM image of the 120 × 30 μm ^2^ device showing the rutile (R) phase formation. The black box at the bottom shows where the DFXM images in (c) and (d) were collected. (b) Voltage–current characteristic of Device 1 (black line) recorded during the DFXM imaging. Red circles correspond to the width of the R-phase filament. The filament width as a function of the applied voltage was extracted from the phase map in panels (c) and (d). (c) R and M1 phase maps obtained in the DFXM experiments through reciprocal space map imaging. An R-phase filament (red area) percolates through the M1-phase matrix (blue area). (d) DFXM phase maps obtained at 7.5, 6.5, 5.5, and 4.5 V. Below the switching threshold (i.e., *V* ≤ 4.5 V) the VO_2_ device is in the M1 phase. (e–g) Extracted **Q** scans from the circled regions in the panel (c). Panel (e) shows a representative M1 domain internal to the filament, panel (f) shows a representative R domain in the filament, and panel (g) shows a representative M1 domain outside the filament. All intensities are scaled with respect to the maximum of the three scans. (h) Extracted M1 domain concentration internal to the filament as the voltage is decreased. The concentration of the M1-domain phase saturates around 20%.

The contrast mechanism of DFXM originates from diffraction, which allows for the spatial differentiation between the rutile (R) and monoclinic (M1) phases in the VO_2_ by collecting images at their respective Bragg conditions. This means that the DFXM measurements are crystal phase (i.e., structure) selective and only probe the regions of the sample that are stoichiometric VO_2_ and diffract as the M1 phase or the R phase. Figure 1b shows the measured resistance–temperature dependence of the VO_2_ sample, which exhibits a first-order MIT with ∼4 orders of magnitude resistance change at *T*_C_ ∼ 340 K. Figure 1c shows the equilibrium (*V* = 0), temperature-dependent X-ray diffraction patterns of VO_2_ that directly shows that the MIT is accompanied by the M1-R structural transition. The separation of the momentum transfer , |Δ**Q**| ∼ 0.012 Å^–1^, between the R (high temperature) and M1 (low temperature) Bragg peaks is significant enough to image the two distinct phases and their spatial distribution that emerges when voltage is applied to VO_2_ devices to trigger the MIT. When studying the phase distribution and evolution at a specific voltage (Figure 2), an image was taken at each position of the scan in Figure 1c. These sets of images were then compiled into a composite image which presents the center-of-mass (COM) of each pixel for the scan.

### Heterogeneous Structure of Filaments

In the first DFXM experiments, the R-phase filament formation within the M1-phase matrix in VO_2_ devices was explored (Figure 2). The filament location was determined by tuning to the (011) R-phase Bragg peak and scanning the larger device (Figure 2a). This was necessary as the increase in flux density, needed for a detectable signal of VO_2_, from the use of the K–B mirrors, reduced the FOV. Once the filament location was determined, DFXM phase maps of the filament region were collected at the black box location in Figure 2a. Phase maps obtained from the DFXM data directly revealed the filament formation in the VO_2_ device under applied voltages (Figure 2c,d). The colors in the phase maps were assigned based on the **Q** value of the center of mass of the spatially resolved diffraction data. The red regions in the maps indicate the presence of the R phase at that location while the blue regions correspond to the M1 phase. At voltage above the threshold, *V*_th_ ∼ 5 V, the phase maps show a clear filamentary state in the device: an R-phase channel is surrounded by the M1-phase matrix.

The filamentary behavior in the DFXM phase maps directly correlates with the *I*–*V* curve; i.e., an R-phase channel is observed when the VO_2_ device is in the low-resistance state at *V* > *V*_th_ (Figure 2b, black curve). A direct correspondence between R/M1 phases and metal/insulator phases, respectively, is expected. DFXM imaging shows that the formation of the R-phase channel is directly responsible for the low-resistance state of VO_2_. When the applied voltage is reduced toward *V*_th_, the width of the filament channel decreases (Figure 2b, red circles), from ∼30 μm at 9.5 V to ∼7 μm at 5.5 V. At *V* < *V*_th_, the R-phase filament disappears, and the device area is predominantly in the M1 phase, which correlates with the switching into the high-resistance state in the *I*–*V* curve. These DFXM observations of the filamentary behaviors are consistent with previous optical studies that reported the electrical switching in VO_2_ to be the result of a metallic phase filament percolating through the insulating matrix.^13,25,27^ Moreover, these DFXM measurements corroborate the nanodiffraction measurements showing an R phase connecting the electrodes in the M1 phase. The filament narrowing with decreasing voltage observed in our DFXM experiment is also in agreement with prior works which also show Joule heating to be the driving transition mechanism.^13,26,47,48^

These measurements further reveal new features in VO_2_ switching devices. Even though the R phase forms the filament, the presence of isolated M1-phase pockets within the filament region: blue M1 spots of 200–300 nm size can be seen within the red R filament in Figure 2c,d in the 5.5–9.5 V range.^49^ Experimental diffraction patterns corresponding to individual regions in the phase maps (a few examples are presented in Figure 2e–g) show very clear differences between the Bragg peaks of isolated M1 spots and R phase filament. It is important to note that due to the film being relatively thick (300 nm), these M1 domains could possibly be beneath the R phase. However, if this were the case then the M1 domains would be expected to populate the entirety of the filament area and signatures of the M1 phase would appear in addition to the R phase signature. This, however, is not what is observed as the signals in the R-phase region only show the R-phase signature (Figure 2f) and these M1 domains only show the M1 signature (Figure 2e). These blue M1 spots within the filament are therefore not due to noise or measurement uncertainty, or stack M1 and R phases vertically. The concentration of these M1 domains internal to the filament can be as high as 20% of the filament area at voltages above the switching threshold (Figure 2h). Reconstructed diffraction peaks from the region inside the filament (Suppl. Figure S1) shows that there the filament region has roughly 25% M1 fraction in good agreement with the area measurements. Temperature-dependent diffraction measurements performed in the same region as the eventual filament (Suppl. Figure S2) show that the M1 phase fraction drops to less than 1% above the temperature driven transition. This indicates that these M1 domains are a feature of the voltage-induced filament and not part of the film’s overall properties.

While the DFXM shows that these domains are in the M1 phase, other research presents different possibilities for the electronic properties of these domains. Previous thermal measurements of the filament reveal that the internal temperature can rise as high as 500 K during the operation with a uniform temperature profile suggesting uniform metallic properties internal to the filament.^47,48^ In conjunction with these thermal measurements, another study has shown the existence of metallic M1 domains during the thermal transition.^8,50^ These studies present the possibility of these observed M1 domains being metallic. However, these studies show that the metallic M1 phase exists only within the transition region. As the DFXM measurements here are only sensitive to structural information, a complete determination of the electronic properties is not possible. Thus, colocated structural and electronic measurements are necessary during the voltage-induced transition to determine the electronic properties of these M1 domains.

### Voltage Cycling Memory in VO_2_

**Figure 3:**
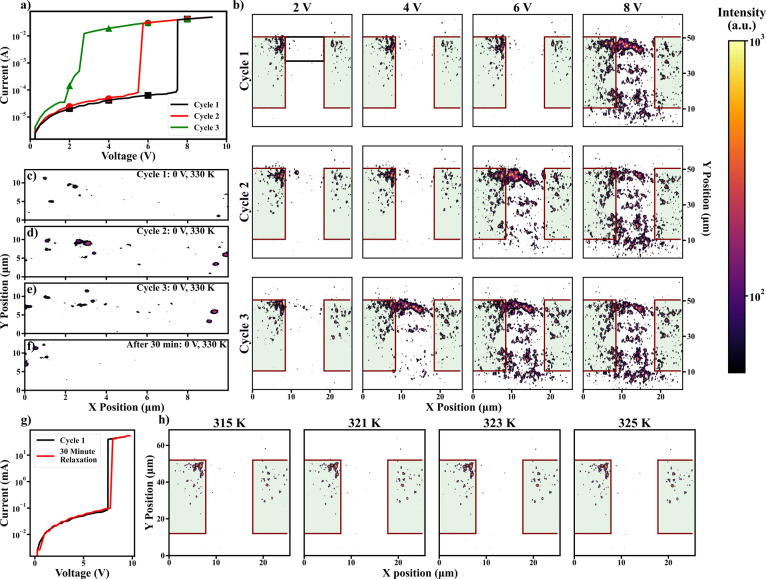
Repeated cycling imaging of a VO_2_ device. (a) Current–voltage characteristic of the VO_2_ device at 330 K. Three electrical cycles are shown. In between the electrical cycles, the device was briefly cooled to room temperature. The points along each curve indicate the voltages at which DFXM images were collected in panel (b). (b) DFXM images were collected at 2, 4, 6, and 8 V for the three consecutive voltage and thermal cycles. The rutile (R) phase gain in the areas below the top of the left electrode appears in all three cycles prior to the filament formation. This R-phase gain determines the preferential pathway of the filament. (c–f) DFXM images within the black box in panel (b) at 0 V and 330 K after performing three consecutive electric and 330 K → 300 K → 330 K thermal cycles. Cycle 1 is shown in panel (c), cycle 2 in panel (d), cycle 3 in panel (e), and after 30 min of relaxation is shown in (f). The sites appear at 330 K with no applied voltage indicating a change into the R phase at a lower *T*_C_. The reduced-voltage domain nucleation demonstrates the emergence of memory upon repeated electrical cycling. (g) *I*–*V* characteristics of cycle 1 (black line) and after a 30 min relaxation (red line). (h) DFXM images showing the formation of the R phase beneath the electrodes (green regions) at temperatures 315, 321, 323, and 325 K.

Enabling short or medium-term memory in VO_2_ could further help achieve practical functionalities such as neuronal adaptation and accommodation operations. In this experiment, the switching cycles were performed at 330 K (i.e., close to *T*_C_) to enable the switching operation using low applied voltages. Between each switching cycle, the device was briefly cooled to room temperature and then heated back to 330 K base temperature (∼10 min per full thermal cycle). This process was used to determine what structural properties influence filament formation location. Electrical measurements showed that VO_2_ devices can maintain information about previous switching events on medium time scales even with a thermal reset (Figure 3a). Even though cooling to room temperature can be expected to completely reset the device phase state (i.e., to convert the VO_2_ volume into M1 phase) a persistent decrease of the threshold voltage upon repeating the switching is observed. During the third cycle, the *V*_th_ is reduced by ∼80%, from *V*_th1_ ∼ 7.5 V to *V*_th3_ ∼ 1.5 V. Similar drastic changes to the threshold voltage have previously been observed in other VO_2_ devices.^51^ However, in these previous experiments, the devices were not cooled into the insulating phase between cycles and thus these voltage reductions are due to R-phase persistence within the device.^25^ Additionally, the V_th_ reduction observed here cannot be attributed to the structural degradation of our VO_2_ devices caused by the voltage cycling as returning the device to room temperature for ∼30 min returned the pristine device behavior (Figure 3g). Therefore, it is concluded that VO_2_ devices can preserve memory about the previous switching events for at least several minutes, even when cooled far below *T*_C_, and resets to the original behavior after tens of minutes.

While electrical measurements directly show a memory effect in VO_2_-switching devices, they do not provide information on the cause of this phenomenon. To explore this memory mechanism associated with repeated switching cycling, DFXM images were taken at the right shoulder of the R-phase Bragg peak, depicted by the gray-shaded region in Figure 1c. At these measurement conditions, the R-phase domains dominate the signal, and the M1 phase contribution is negligible. Thus, these measurements selectively identify the spatial locations of the R-phase domains within a few seconds of the X-ray exposure. When the device was briefly cooled to room temperature between the electrical cycles, the R-phase domains were fully reset into the M1 phase as confirmed by acquiring DFXM images in 315–325 K temperature range (Figure 3h). This indicates that the observed cycle-to-cycle reduction of V_th_ is not directly related to the R-phase persistence, which potentially could occur if the device was not thermally cycled. However, it was found that during each cycle, the voltage-induced R-phase filament forms at approximately the same location, close to the top of the device in Figure 3b. While the general filament appearance is nearly identical between the cycles, the R-phase domains tend to cluster near the prior location of the filament with each consecutive electrical cycle (i.e., a smaller number of isolated R-phase pockets nucleate outside the filament). A comparison of the zoomed-in region where the filament forms can be seen in Figure 3c–f. These panels correspond to the region indicated by the black box on the top left panel in Figure 3b. The images in Figure 3c–f were acquired at zero voltage and 330 K, right before performing a switching cycle. Even with no bias voltage, there are regions that have already transitioned into the R phase (Figure 3c–e). This indicates that these regions have been modified, by the percolation of the filament, to have a *T*_C_ below 330 K. Comparing the images acquired after consecutive cycles (Figure 3c–e), more R-phase clusters populate the region along the filament line after each switching cycle, further promoting filament growth along the same direction. Finally, after a 30 min relaxation at room temperature, the appearance of these lower *T*_C_ regions vanished (Figure 3f), effectively resetting the memory of the device as observed from the electrical measurements (Figure 3g). It is important to note that, unlike previous nanodiffraction studies,^24^ due to the reduction in the collection time, the measurements here were able to observe this medium-term memory and its origin.

In conjunction with these sites forming within the device gap, R-phase clusters are also observed beneath the electrodes prior to the filament formation (Figure 3b,g). This local R-phase accumulation, within the proximity of the eventual filament formation location, suggests that the R-phase clusters under the electrodes may serve as a precursor event to the MIT. This further explaining this memory phenomenon and filament pinning (Figure 3b). Previous nanodiffraction measurements showed that the filament in VO_2_ switching devices can partially extend under the electrode.^24^ Likely because of the small FOV in the nanodiffraction, it was concluded that proximity to the hot filament is responsible for causing the R phase formation under the electrodes. Owing to the wide FOV in our DFXM measurements, the R phase extends a very large distance under the electrode, over a device gap, where the heating effect of the filament should be insignificant (Figures 2a and 3b). DFXM maps taken at temperatures as low as 315 K under no voltage reveal a reduced *T*_C_ beneath the electrodes (Figure 3h). This lower *T*_C_ can be a result of the electrode fabrication procedures. For example, a slight chemical composition change can be due to the Ti adhesion layer in the Ti/Au electrodes or these electrodes may cause local lattice distortion.

### Resistor Network Simulations

**Figure 4:**
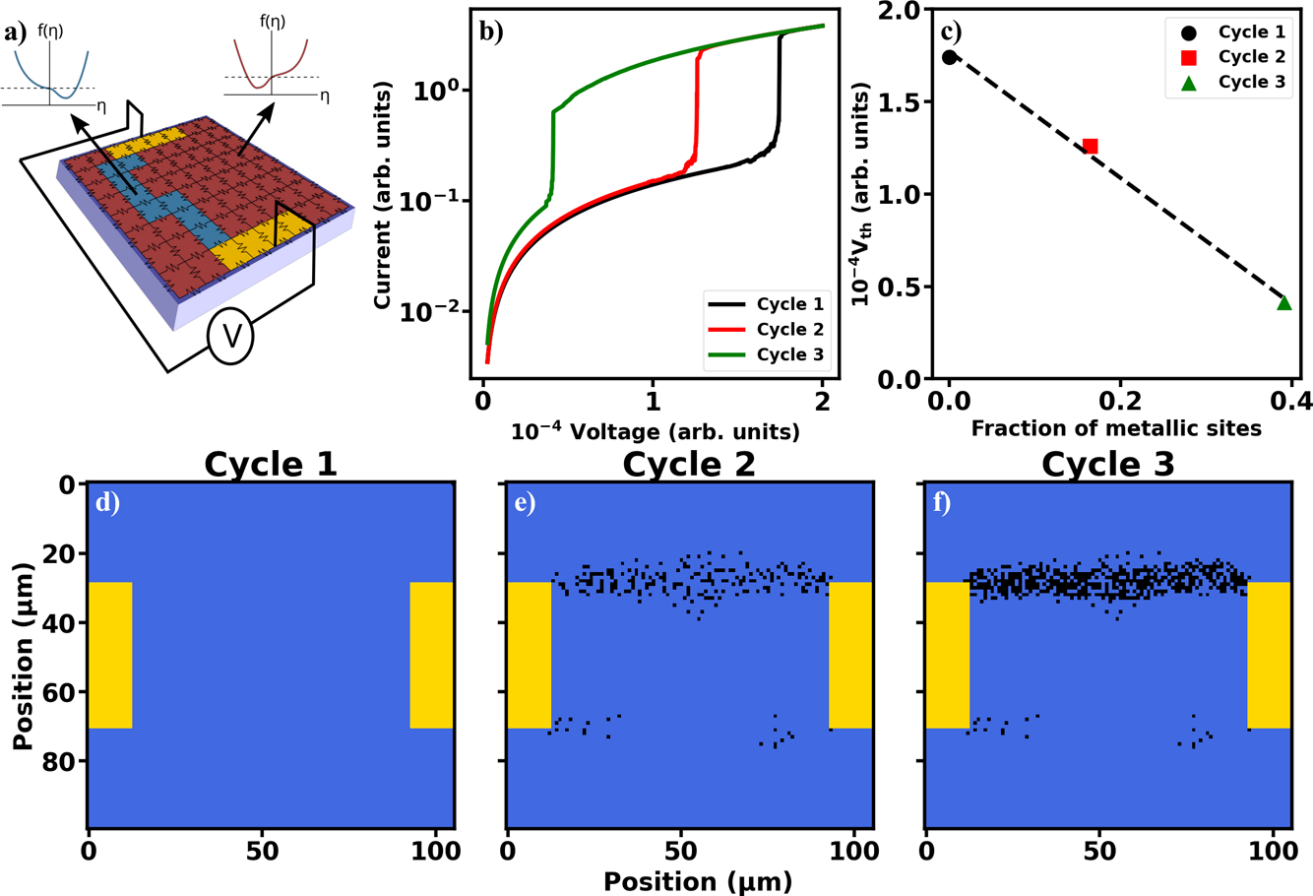
Simulations of cycling and memory formation. (a) Schematic of the Mott resistor network. (b) Simulated *I*–*V* characteristics for the three cycles. (c) Reduction in the threshold voltage with increasing metallic domains at 330 K along the filament formation region. (d–f) Distribution of metallic domains in the simulated device with the temperature set to 330 K and 0 V for the first cycle (d), second cycle (e), and third cycle (f). Blue regions show insulating phases, black spots show metallic, low-*T*_C_ sites, and yellow areas correspond to the electrodes.

To gain insight into the physical mechanisms responsible for the experimentally observed switching cycle memory (Figure 3), numerical calculations were performed using a Mott resistive network.^25,52^ A schematic of the resistor network model is shown in Figure 4a. Each resistor in the network can be either in an insulating or metallic state. The first order MIT of VO_2_ was modeled using a Landau type free energy functional (see refs (25 and 52)). In the simulations, neighboring cells interact through thermal effects and current. At each applied voltage, the resistor network is solved to obtain local voltage/current distribution and local Joule heating to compute local temperatures. Depending on the local temperature, each resistor in the network can undergo the MIT. After updating the phase state of each resistor, the computing process is repeated (solve for the local voltage/current distribution, obtain local temperature, update local phase state) until all variables converge.

To model the memory effect associated with repeated switching cycling, a small probability was introduced such that individual resistors in the network become stuck in a state with a lower phase transition temperature when undergoing a switching cycle. This low-*T*_C_ switching probability was introduced phenomenologically in the simulations following the experimental observations of R domains’ appearance after each switching cycle. The switching probability was tuned such that the relative voltage threshold decrease matched that observed in the experiments (Figure 3a). A probability of ∼0.09% was found to be appropriate to model the *I*–*V* characteristics. Subjecting the network to multiple switching cycles results in the accumulation of these low-*T*_C_ resistors along the region where the filament formed during the switching (Figure 4d–f). These low-*T*_C_ sites display a tendency to cluster closer together with each consecutive switching cycle (compare panels Figure 4e,f). This is due to these low-*T*_C_ resistors attracting the filament formation and, consequently, new low-*T*_C_ sites appear in the vicinity of the old ones due to the small probability of a reduction in *T*_C_ for metallic cells. The simulation results agree well with the experimental observation of the accumulation of R-phase domains inside the switching device after each switching cycle and the tighter clustering of the switched filament (Figure 3b–e). Moreover, these low-*T*_C_ sites have a dramatic impact on the *I*–*V* characteristic of the switching device (Figure 4b): the threshold voltage and the initial, low-voltage resistance of the entire device decrease substantially after each consecutive switching cycle, in good correspondence with the experimental observations (Figure 3a). The simulations show that ∼40% of individual low-*T*_C_ point defects inside the filament area can reduce the threshold voltage by ∼75% (Figure 4c) and entice the filament formation to a particular location within the device.

Although the origin of such local *T*_C_ modulations is unknown, local low-*T*_C_ site formation in our devices could be related to the ramp-reversal memory observed in several MIT materials, including VO_2_.^28,29^ In ramp-reversal experiments, an MIT sample is subject to thermal cycling and each cycle produces a slightly different resistance state. Optical mapping of the ramp reversal memory state has revealed the emergence of both low- and high-*T*_C_ clusters inside the material.^29^ Assuming that in our experiments the MIT switching is due primarily to Joule heating, our voltage cycling process can be compared to thermal cycling in ramp reversal experiments. Therefore, low-*T*_C_ sites can emerge inside our VO_2_ device as each voltage cycle raises and then lowers the local temperature.

## Conclusions

The large FOV and short signal acquisition time of DFXM enabled the observation of unique structural features of the MIT switching in VO_2_ devices. It was found that (i) the R phase forms in the large extended areas beneath the electrodes prior to device switching, (ii) the filament is not structurally uniform with small M1-phase pockets appearing in the filament, and (iii) MIT switching induces a medium-term memory upon repeated electrical cycling resulting in the nucleation of R-phase domains at applied voltages much lower than the switching threshold further reinforcing the filament formation along a preferential pathway. The memory effect persists even after a short thermal reset of the device, while a longer thermal reset (30 min), erases the memory, indicating that the memory cannot be directly attributed either to the first-order thermal hysteresis or electrically induced nonvolatile ionic migration. Further studies are necessary to understand the physical origin of this medium-term memory. The identification of a preferential pathway suggests that local structural modulation could be used to entice the filament formation in a desired place and can help tune switching voltages. Such modulation could be engineered, for example, using laser-etching, local hydrogen doping, or focused ion beam irradiation.^36,53^ The formation of the R phase beneath the electrodes appearing before the device switching and extending well beyond the local thermal influence of the filament indicates a complex interaction between the electrodes and film. The proposed mechanism of modified *T*_C_ defects producing a reduction in the threshold voltage matches well with the observed microscopy data and electrical measurements. Thus, an accumulation of conducting, low-*T*_C_ defects, through a ramp-reversal memory mechanism, could likely be responsible for the observed medium-term memory upon repeated switching cycling. With the continued development of DFXM at upgraded modern synchrotron radiation sources, imaging structural dynamics at time scales of milliseconds and faster may be possible. With the continuing development of the synchrotron radiation sources and detector capabilities, observations of the structural dynamics at time scales of milliseconds and below using DFXM imaging may become possible.^54,55^ Our results obtained in VO_2_ thin-film devices also provide a benchmark for the DFXM studies of other weak-scattering phenomena, such as scattering from magnetic features and charge density wave domains.

## Methods/Experimental Section

### Dark-Field X-ray Microscopy

The dark-field X-ray microscopy (DFXM) measurements were performed at 6-ID-C and 33-ID-D beamlines of the Advanced Photon Source at Argonne National Laboratory.^39,56−58^Figure 1a shows the DFXM setup that was used to explore local structural changes during the electrically induced filament formation in VO_2_ devices. A Fresnel Zone Plate (FZP, was utilized 100 μm diameter and 70 nm outer zone width) was utilized with a zeroth order block (not shown) as an objective lens that had a focal length of 56.5 mm at 10 keV. With a sample-to-scintillator distance of 1470 mm, an overall X-ray magnification *M*_X-ray_ ∼ 24 was achieved. The short focal length offered by the FZP allows for the high magnification and resolution necessary to image nanoscale domains of the monoclinic (M1) and rutile (R) phases in VO_2_. An additional optical magnification *M*_O_ = 20 coming from an optical lens placed between the X-ray scintillator and optical detector further enhanced the total magnification to ∼480 (Figure 1a). Overall, the effective pixel size was 15 nm with a resolution of approximately 70 nm, comparable to the resolution of the state-of-the-art nanodiffraction setups.^24^

The measurements at 33-ID-D were performed using 10 keV X-ray energy. Measurements were performed in a symmetrical reflection geometry with an incident angle of ∼14.8° (VO_2_ (011)_R_ Bragg peak, (100)_M1_ Bragg peak) resulting in a beam footprint (i.e., the FOV) of ∼30 × 120 μm^2^. The use of a reflection geometry reduced our resolution along the X-ray direction by a factor of 4–300 nm. It is important to note that no discernible X-ray damage due to the increased flux density was observed in the repeatability of the device operation.

### Samples

The VO_2_ sample used in this study was a 300 nm-thick film. The film was grown by the reactive radio frequency (RF) sputtering using a stoichiometric V_2_O_3_ target. Details about the film growth and device preparation can be found elsewhere.^13^ The VO_2_ phase formation and transformation was confirmed by the specular X-ray diffraction measurements (Suppl. Figure S3b and S4) and film thickness was determined by calibrating the growth procedures. The electrodes were comprised of (20 nm Ti)/(100 nm Au), defining planar two-terminal devices which were prepared using the standard optical lithography and e-beam evaporation. Resistance–temperature measurements showed an abrupt first-order phase transition at *T*_C_ ∼ 340 K with approximately 4 orders of magnitude resistance change (Figure 1b). Importantly, the resistance–temperature curves of the film before and after device fabrication were identical (Suppl. Figure S3a), which indicates that the device preparation procedures did not alter the VO_2_ film’s stoichiometry or structure. Saturation into the insulating and metallic states of the device earlier than the film is likely due to the larger area between the electrical contacts in the film probing a larger area of the sample. An SEM image of the VO_2_ film unpatterned (Suppl. Figure S5) indicates the average domain size to be 300 nm. In the DFXM experiments, two devices were studied: a 30 × 120 μm^2^, Device 1, and a 10 × 40 μm^2^, Device 2.

### Mott Resistor Network

The simulations performed in this article are based on the Mott resistor network model.^59^ The VO_2_ sample is described as a two-dimensional grid of cells, where each cell represents a nanoscale region of the sample and contains four resistors. These resistors connect each cell, and the resistance value of each resistor depends on the local temperature. To model the first-order character of the insulator–metal transition in VO_2_, the stability of the metal and insulator phases of each resistor depends on the local temperature via a Landau-type free energy functional.^60^ For the simulations performed within this article, the Mott resistor network consists of a 100 × 106 grid of cells. When a voltage is applied across the grid, currents start flowing through the resistors, locally generating heat per Joule’s first law *P* = *IV*. The resistor network is assumed to be in thermal contact with a perfectly insulating substrate that is held to a fixed temperature. The local temperature of each cell is equal to the sum of the Joule heating contribution and the heat exchanged between the neighboring cells and the substrate. Once the temperature of a cell is computed, it is used to update the resistance value of the four resistors within. The network is connected to the rest of the circuit representing the experimental setup by two ideally metallic electrodes located at the top and bottom of the grid. The resistance of the network collapses when a percolating group of cells undergoes an insulator–metal transition, forming a highly conducting filament between the electrodes.

When modeling the memory effect in these simulations, a small probability was introduced that a cell could lower its transition temperature below 330 K. This probability was applied only when a cell was in the metallic state. This parameter was tuned such that the percent changes to the threshold voltage after each cycle matched those observed experimentally. The probability that matched these changes was 0.089129% per time step. The model is highly sensitive to this parameter with higher probabilities resulting in complete metallic behavior and smaller probabilities resulting in smaller threshold voltage changes. Due to the increased electric fields at the corners of the electrodes, filaments prefer to form near the corners. The initial filament formation between one of the two corners is determined by a temperature-based probability of transitioning between the insulating and metallic states. This spontaneously breaks the symmetry, producing a filament between either the top or bottom corners. After the first cycle, the metallic defects maintain this broken symmetry further reinforcing filament formation in the same locations.

## Supporting Information Available

Further analysis of the monoclinic domains internal to the filament; temperature dependent X-ray diffraction in the region of filament formation; device operation information; characterization of samples including RT measurements, XRD, SEM images of the film and IR image of the filament; and data processing information on calculation of the center of mass compiled images (PDF)

## Notes

This manuscript has been previously submitted to a preprint server. The preprint version of this work is cited as follows: E.K.; P.S.; I.P.; et al. High-Resolution Full-field Structural Microscopy of the Voltage Induced Filament Formation in Neuromorphic Devices. 2023, preprint, arXiv. 10.48550/arXiv.2309.15712 (accessed March 31, 2025).
