# Supplementary material for: High-Resolution Full-Field Structural Microscopy of the Voltage-Induced Filament Formation in VO2-Based Neuromorphic Devices
Source: ACS Nano. 2025 Apr 14;19(16):15385–94. doi: 10.1021/acsnano.4c14696 (PMC12044682; doi:10.1021/acsnano.4c14696)
Supplement: Supplementary file 1 — nn4c14696_si_001.pdf [file nn4c14696_si_001.pdf]

Supp. Mat.: High-resolution full-field structural microscopy of the voltage-induced filament formation in VO_2-based neuromorphic devices

Elliot Kisiel,[*,†,‡] Pavel Salev,[¶] Ishwor Poudyal,[‡,§] David J. Alspaugh,[†] Fellipe Carneiro,[⊥,#] Erbin Qiu,[†] Fanny Rodolakis,[‡] Zhan Zhang,[‡] Oleg G. Shpyrko,[†] Marcelo Rozenberg,[†,∥] Ivan K. Schuller,[†] Zahir Islam,[‡] and Alex Frano[*,†,@]

†*Physics Department, University of California San Diego, La Jolla, CA 92093, USA.*

‡*X-ray Science Division, Argonne National Laboratory, Lemont, IL 60439, USA*

¶*Department Physics and Astronomy, University of Denver, Denver, CO 80210, USA.*

§*Materials Science Division, Argonne National Laboratory, Lemont, IL 60439, USA*

∥*Laboratoire de Physique des Solides, CNRS-UMR 8502, Université Paris-Sud, Orsay 91405, France*

⊥*Materials Physics and Applications, Los Alamos National Laboratory, Los Alamos, New Mexico 87544, USA.*

#*Centro Brasileiro de Pesquisas Físicas, Rio de Janeiro, RJ 22290-180, Brazil.*

@*Program in Materials Science and Engineering, University of California San Diego, La Jolla, CA 92093-0418, USA*

E-mail: ekisiel@anl.gov; afrano@ucsd.edu

Diffraction from Thermally-Driven Transition

The appearance of M1 domains internal to the filament has not been previously reported in the literature. To confirm that these domains are a feature of the filament and not the film, temperature-dependent diffraction measurements of the film in the region where filaments form were performed. These diffraction measurements were performed with the same beam size in the same region as the filament allowing for a direct comparison of the diffraction measurements and DFXM maps. Comparing the diffraction measurements with those from DFXM maps, it is identified that these M1 domains are a feature of the filament not inherent to the film. Fig. S2 shows the diffraction spots for varying temperatures through the transition. Below the transition, the film is entirely in the M1 phase and gradually transforms into the R phase. After the transition, the region is entirely in the R phase ($< 1\%$ M1 regions) much less than the 20% observed internal to the filament. Fig. S1 shows the comparison of the diffraction peaks of the filament region with those observed from the diffraction measurements as a function of temperature. The phase fraction of the M1 phase internal to the voltage-induced filament ($\sim 25\%$) agrees well with the 20% M1 domain measure from the area measurements of the M1 domains. It is noticeable that there is a distinct lack of M1 signal above the transition temperature (Fig. S1d).

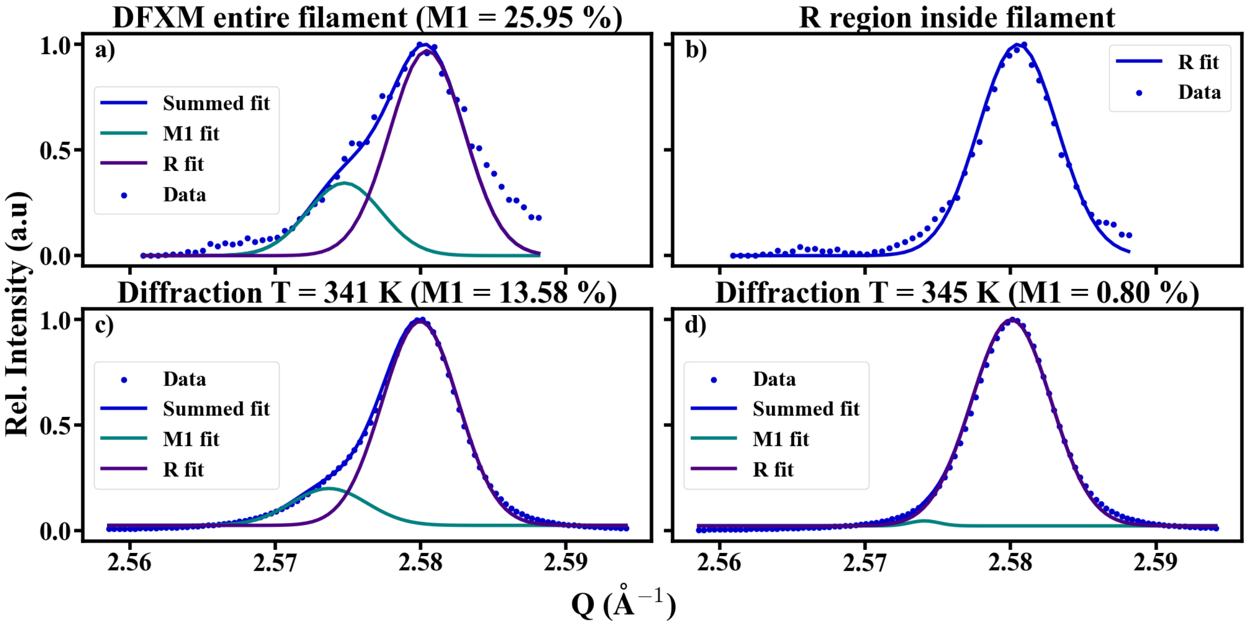

Figure S1: a) The diffraction curve extracted from a DFXM map internal to the filament at 10 V. There is a noticeable shoulder that is not present in panel (b). b) Diffraction curve extracted from DFXM map in a region that shows solely only R phase presence internal to the filament. c) Diffraction curve from the temperature-dependent diffraction measurement collected at 341 K showing $\sim$ 13% M1 phase fraction. d) Diffraction curve from the temperature-dependent diffraction measurement collected at 345 K showing $\sim$ 1% M1 phase fraction.

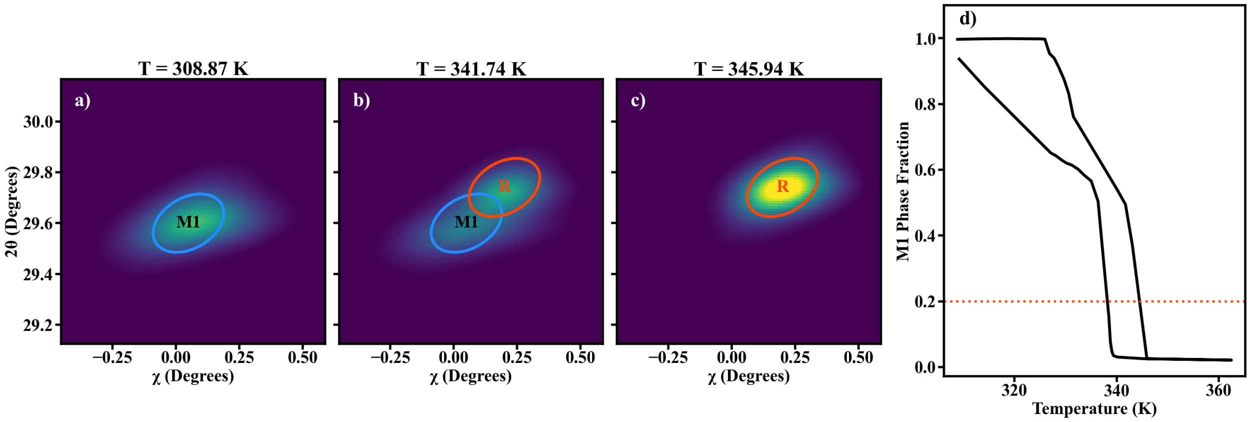

Figure S2: a-c) The M1 and R peaks moving through the transition of 343 K. The M1 and R peaks are labeled. d) The M1 fraction as a function of temperature. The red line indicates the 20% area fraction observed from the DFXM measurements of voltage-induced filament. It is noticeable that above the transition temperature, the M1 phase fraction is less than 1%.

Device Cycling

A voltage source was used to induce the MIT and a 150 Ω resistor was connected in series with the VO_2 device to limit the current. The filament formation was achieved by utilizing an electric field cooling (EFC) protocol. First, the sample was heated to 350 K, i.e., above T_c, and 10 V was applied. Then the sample was cooled down to room temperature while maintaining the applied voltage. Finally, DFXM imaging was performed at room temperature while ramping the voltage down to zero. Employing the EFC protocol helped avoid accidental electrical breakdown of the VO_2 device, which was instrumental in conducting the first tests of the new imaging technique.

Film Growth and Characterization

300-nm-thick VO_2 film was grown by the reactive rf sputtering using a stoichiometric V_2O_3 target. The substrate temperature during the growth was 460°C. The growth was done in 3.4 mTorr 92% Ar and 8% O_2 atmosphere. The rf power was 100 W resulting in a ~3.2 nm/min growth rate. After the growth, the sample was cooled down at 12°C/min to room temperature while maintaining the Ar/O_2 atmosphere. To isolate individual devices on the same chip, optical lithography and reactive ion etching were used to pattern the 400×400 µm^2 VO_2 islands surrounding each device. The reactive ion etching was done in 50 mTorr 17% Ar and 83% Cl_2 atmosphere at 200 W rf power giving a ~100 nm/min etching rate.

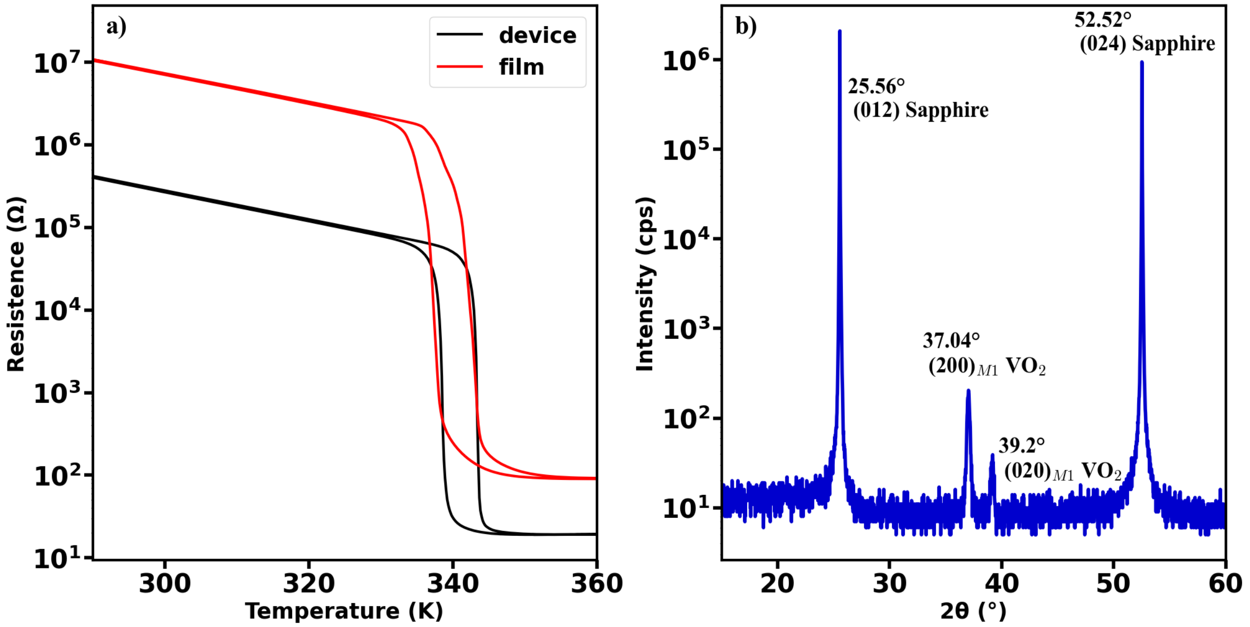

Figure S3: a) The resistance as a function of temperature showing the hysteresis of the VO_2 film (red) and etched device (black). b) Preliminary specular XRD measurements were taken on the unpatterned film performed at room temperature using a Cu-Kα source.

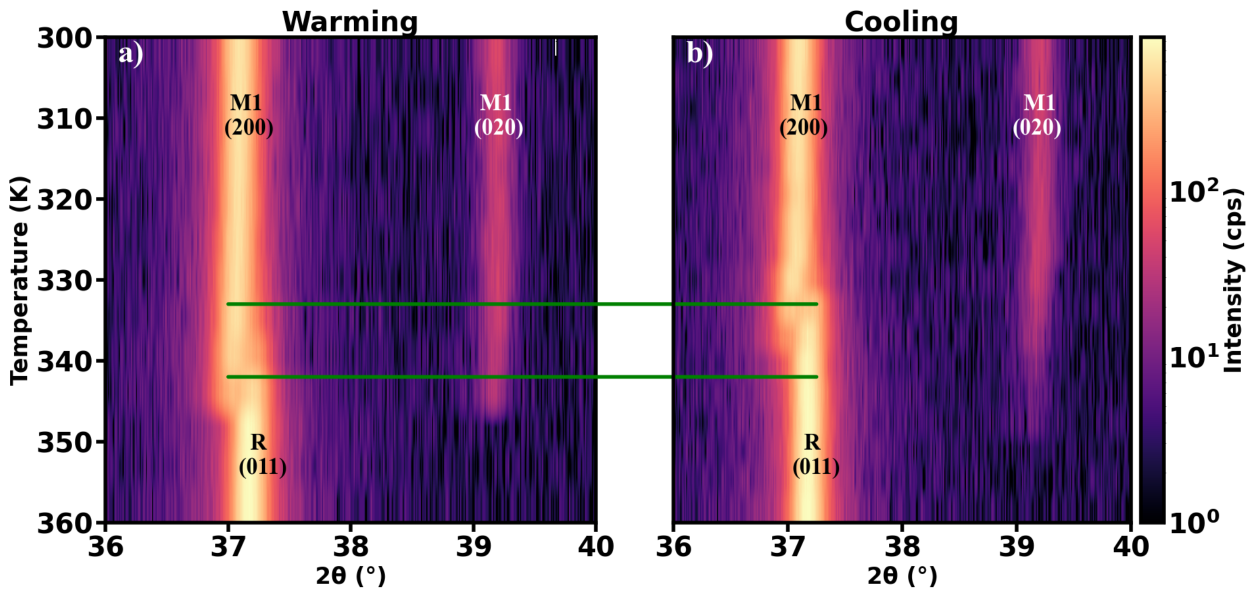

Figure S4: a) Mapping of the warming cycle of the unpatterned film showing the temperature progression of the phase transformation. b) Mapping of the cooling cycle of the unpatterned film showing the temperature progression of the phase transformation. The green lines indicated the temperature width for this transition's hysteresis. The phases labeled correspond to the (200) peak for the M1 phase and the (011) for the R phase. The peak to the right is the (210) monoclinic peak which travels out of alignment as temperature increases.

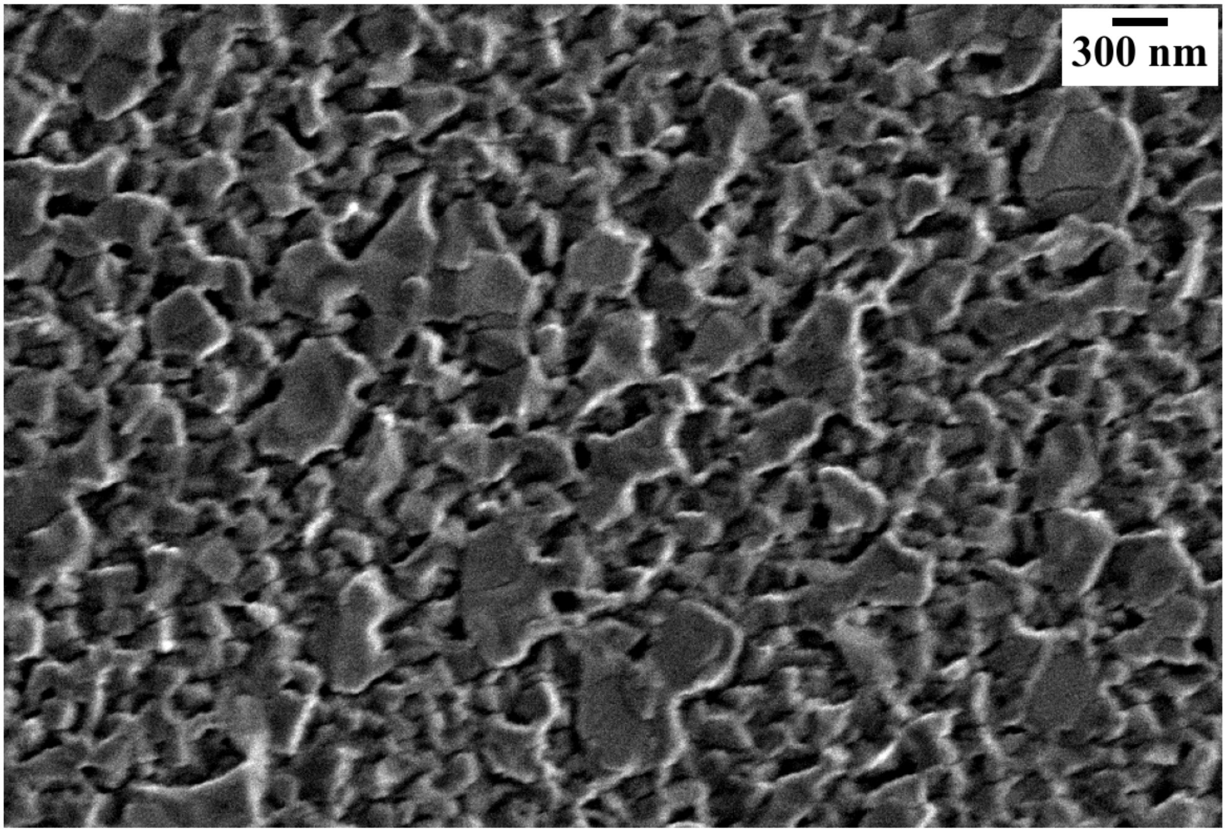

Figure S5: An SEM image taken at 300 K of an unpatterned VO_2 film showing the domain sizes to be on the order of 300 nm.

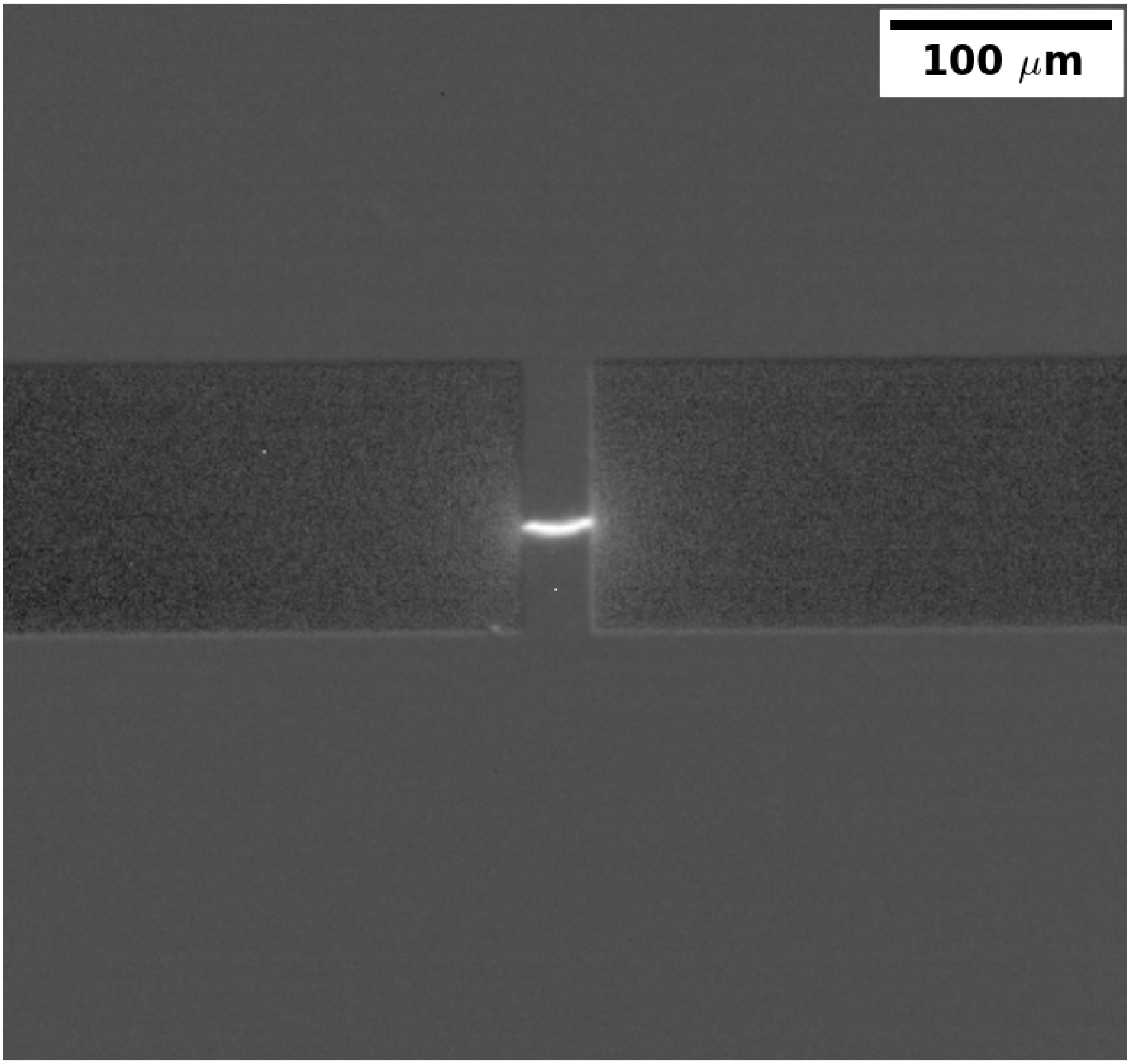

Figure S6: An IR image showing the filament formation in the 30 x 120 μm^2 VO_2 device.

Heating Stage

A heating stage using a Peltier heater was used to heat the sample close to the transition temperature. Previous measurements on the film in air showed no electrical differences when the sample was left above the transition temperature. Characterization of the heater showed that it was stable to ± 0.5 K over the course of 1 hour which is well below the time per imaging cycle.

Image Processing Procedure

Detector images were collected in 16-bit tif file and collected for every imaging scan performed. A background subtraction using a background estimator of a biweight background estimator as a robust measure of the background. This method gave a consistent background with dark images collected during one of the scans. To remove hot pixels, we utilized a median filter with a kernel size of 5. A bottom threshold application was also applied to remove any additional backgrounds missed near the edge of the detector. Images were converted to edf files for use with the *darfix* package.[1] The center of mass per pixel is calculated via,

$$Q_\mu = \frac{\mathbf{I} \cdot \mathbf{Q}}{\sum_{i=0}^{k} I_i} \tag{1}$$

Where $\mathbf{I}$ is a 1-D vector of the intensity for a single pixel per scan, $\mathbf{Q}$ is the 1-D vector of the measured Q values and Q_μ is the calculated center of mass position.

References

1. Garriga Ferrer, J.; Rodriguez-Lamas, R.; Payno, H.; De Nolf, W.; Cook, P.; Sole Jover, V. A.; Yildirim, C.; Detlefs, C. darfix - data analysis for dark-field X-ray microscopy. *Journal of Synchrotron Radiation* **2023**, *30*, 527–537.
